# Retraction Note: Exosome-transmitted miR-128-3p increase chemosensitivity of oxaliplatin-resistant colorectal cancer

**DOI:** 10.1186/s12943-022-01627-4

**Published:** 2022-07-30

**Authors:** Tong Liu, Xin Zhang, Lutao Du, Yunshan Wang, Xiaoming Liu, Hui Tian, Lili Wang, Peilong Li, Yinghui Zhao, Weili Duan, Yujiao Xie, Zhaowei Sun, Chuanxin Wang

**Affiliations:** 1 Department of Clinical Laboratory, The Second Hospital of Shandong University, No. 247 Beiyuan Street, Jinan, 250033 China; 2 Department of Clinical Laboratory, Qilu Hospital, Shandong University, Jinan, 250012 Shandong Province China; 3 Department of Preventive Medicine, Shandong Provincial Traditional Chinese Medical Hospital, Jinan, 250012 People’s Republic of China; 4 Cancer Center, Qilu Hospital, Shandong University, Jinan, 250012 Shandong Province China; 5 Department of Surgery, The Affiliated Hospital of Medical College Qingdao University, Qingdao, 266071 Shandong Province China

**Retraction Note: Mol Cancer 18, 43 (2019)**

**https://doi.org/10.1186/s12943-019-0981-7**

The Editor in Chief has retracted this article because of significant concerns regarding figures 2F, 2G, 5D, 6G and additional file 10 presented in this work, which question the integrity of the data. Concerns have also been raised regarding methods used in this article, specifically the isolation of the exosomes, the animal experiment controls and the cell culture. The Editor in Chief, therefore, no longer has confidence in the presented results and the conclusions drawn in this study. None of the authors have responded to any correspondence from the editor/publisher about this retraction.

